# Intraoperative hyperglycemia is independently associated with infectious complications after non-cardiac surgery

**DOI:** 10.1186/s12871-018-0546-0

**Published:** 2018-07-19

**Authors:** Amy M. Shanks, Derek T. Woodrum, Sathish S. Kumar, Darrell A. Campbell, Sachin Kheterpal

**Affiliations:** 1 Department of Anesthesiology, Michigan Medicine, 1500 E. Medical Center Dr., SPC 5048, Ann Arbor, MI 48109 USA; 2 Department of Surgery, Michigan Medicine, 1500 E. Medical Center Dr., SPC 5825, Ann Arbor, MI 48109 USA

**Keywords:** Hyperglycemia, Infection, Surgery

## Abstract

**Background:**

Perioperative hyperglycemia and its associated increase in morbidity and mortality have been well studied in the critical care and cardiac surgery literature. However, there is little data regarding the impact of intraoperative hyperglycemia on post-operative infectious complications in non-cardiac surgery.

**Methods:**

All National Surgery Quality Improvement Program patients undergoing general, vascular, and urological surgery at our tertiary care center were reviewed. After integrating intraoperative glucose measurements from our intraoperative electronic health record, we categorized patients as experiencing mild (8.3–11.0 mmol/L), moderate (11.1–16.6 mmol/L), and severe (≥ 16.7 mmol/L) intraoperative hyperglycemia. Using multiple logistic regression to adjust for patient comorbidities and surgical factors, we evaluated the association of hyperglycemia with the primary outcome of postoperative surgical site infection, pneumonia, urinary tract infection, or sepsis within 30 days.

**Results:**

Of 13,954 patients reviewed, 3150 patients met inclusion criteria and had an intraoperative glucose measurement. 49% (*n* = 1531) of patients experienced hyperglycemia and 15% (*n* = 482) patients experienced an infectious complication. Patients with mild (adjusted odds ratio 1.30, 95% confidence interval [1.01 to 1.68], *p*-value = 0.04) and moderate hyperglycemia (adjusted odds ratio 1.57, 95% confidence interval [1.08–2.28], *p*-value = 0.02) had a statistically significant risk-adjusted increase in infectious complications. The model c-statistic was 0.72 [95% confidence interval 0.69–0.74].

**Conclusions:**

This is one of the first studies to demonstrate an independent relationship between intraoperative hyperglycemia and postoperative infectious complications. Future studies are needed to evaluate a causal relationship and impact of treatment.

**Electronic supplementary material:**

The online version of this article (10.1186/s12871-018-0546-0) contains supplementary material, which is available to authorized users.

## Background

Postoperative hyperglycemia has been associated with increased risks of complications among patients undergoing cardiac surgery and critically ill patients [1–5]. In particular, hyperglycemia is thought to decrease the body’s immune response and its ability to fight bacterial infection [1, 5, 6]. However, attempts to demonstrate the value of intensive insulin therapy (IIT) have had mixed results, with some studies demonstrating that the risk of hypoglycemia outweighs possible benefits of normoglycemia [6–9]. As a result, even studied populations have had evolving and often contradictory glucose control standards over the last decade.

There is a paucity of intraoperative data regarding patients undergoing non-cardiac surgery with no compelling data guiding the optimal glucose target during or after the operation. Most observational or interventional studies evaluating hyperglycemia and non-cardiac surgery have focused on preoperative or postoperative glucose measurements due to challenges in obtaining intraoperative measurements manually entered into paper or electronic anesthesia records [10–15]. Practicing anesthesiologists have no data to guide their intraoperative glycemic management, resulting in wide variation of treatment triggers and definitions of ‘tight’ control. Two recent randomized controlled trials focused on vascular surgery patients have had conflicting results, with one observing decreased morbidity and mortality associated with tight glucose control, while the other trial failed to observe a statistically significant difference [16–18]. Recently, a prospective study designed to electronically remind clinicians intraoperatively to recheck glucose values on diabetic patients without any specific glucose treatment goals documented a reduced relative risk reduction of 55.4% in surgical site infections [19]. However, no target glucose ranges for normoglycemia were required and the actual achieved intraoperative glucose values were not reported. A review of the national trial registry databases in both the United Kingdom and United States of America currently found no actively recruiting clinical trials evaluating the relationship between intraoperative glucose thresholds for treating and postoperative infections.

The purpose of the current study was to evaluate the relationship between intraoperative glucose levels and postoperative outcomes in non-cardiac surgery. In particular, evaluating the impact on infectious complications may drive a more specific analysis based upon the pathophysiology of hyperglycemia [13–15]. We hypothesized that patients undergoing major non-cardiac surgery and demonstrating intraoperative hyperglycemia would experience elevated rates of infectious complications. By integrating prospective risk adjustment and 30-day outcome data from the National Surgical Quality Improvement Program (NSQIP) with intraoperative electronic health record (EHR) glucose measurements, a novel database of thousands of patients may inform future studies of hyperglycemia in non-cardiac surgery.

## Methods

Institutional review board approval (University of Michigan, Ann Arbor, Michigan) was obtained for this retrospective observational study at a single academic medical center. As there were no care interventions involved and protected health information were removed after the dataset was created, patient consent was waived as part of the approval.

### Patient population

All adult patients (age ≥ 18 years) with or without a diagnosis of diabetes mellitus undergoing elective and emergency general, vascular, or urologic procedure were included in this study. Data was extracted from NSQIP data collection process and intraoperative electronic health records at our center from 2005 to 2010.

Patients with a scheduled outpatient procedure were excluded due to low expected infectious complication rate. Additional exclusion criteria were: American Society of Anesthesiologists Physical Status classification (ASA) 5 or 6, a preoperative systemic inflammatory response syndrome, sepsis, septic shock, pneumonia, preoperative wound classification of dirty, and confirmed pregnancy. Finally, patients without any glucose measurements during the intraoperative period were also excluded.

### Data collection

Data were collected by integrating the local NSQIP database with the departmental perioperative EHR, Centricity® (General Electric Healthcare, Waukesha, Wisconsin). The NSQIP methodology has been described previously [20, 21]. For each operation, a trained surgical clinical quality reviewer collects patient demographics, preoperative comorbidities, operative informative, and postoperative adverse occurrences up to 30 days after the operation. Inter-rater reliability data checks are routinely performed and any disagreements > 5% are investigated. Detailed definitions of NSQIP preoperative patient demographics and comorbidities have been described previously and are available in Additional file 1: Appendix 1 [20, 21].

Intraoperative glucose measurements were obtained from the EHR. This included data from the enterprise laboratory information system and values entered manually by the anesthesiology provider using point of care testing which includes, testing using Accu-Check® (Roche Diagnostics, Indianapolis, Indiana), point of care blood gas analysis RapidPoint 400® (Siemens Medical Solutions, Malvern, Pennsylvania), and formal laboratory testing values. The Accu-Check® point of care device has previously been shown to have an acceptable accuracy over a wide range of hematocrit values in critically ill patients [22]. The EHR data and NSQIP data were linked using patient medical record number combined with anesthesia start date and time using an honest broker system [23]. The use of intraoperative insulin therapy, either intravenous or subcutaneous, as a bolus or infusion, was extracted from the EHR. Once these data were linked, patient identifiers were removed from the analytical dataset. Glucose measurements during the “anesthesia period”, defined as anesthesia start to anesthesia end, were included in the analysis. For each period, all of the glucose measurements were retrieved for each patient in order to derive five distinct summary measures: minimum, maximum, median, standard deviation, and glycemic lability index (GLI). The GLI is adapted from the critical care literature and has been demonstrated to be an important measure of glycemic variability [24]. It is calculated as the squared difference between consecutive glucose measures per unit of actual time between those samples and corrected for variant number of measurements and observation time. Specifically, the GLI was calculated as:

$$ {\displaystyle \begin{array}{c}\sum \left[{\left({\mathrm{Glu}}_{\mathrm{n}}-{\mathrm{Glu}}_{\mathrm{n}+1}\right)}^2\ast \left({\mathrm{Time}}_{\mathrm{n}+1}-{\mathrm{Time}}_{\mathrm{n}}\right)\kern0.5em \left.\mathrm{in}\kern0.5em {\mathrm{minutes}}^{-1}\right)\ast \right.\\ {}\left.{\left(\mathrm{number}\ \mathrm{of}\ \mathrm{measurements}\right)}^{-1}\ast {\left(\mathrm{observation}\ \mathrm{time}\ \mathrm{in}\ \mathrm{hours}\right)}^{-1}\right]\end{array}} $$

Given the lack of substantial evidence regarding intraoperative hyperglycemia and outcomes in non-cardiac surgery, no existing definitions of elevated glucose levels during surgery were available in the peer-reviewed literature. For the purposes of this analysis, normoglycemia was defined as a maximum glucose of less than 8.3 mol/L. Using the Delphi Method, mild, moderate, and severe hyperglycemia was defined as a maximum glucose measurement of between 8.3–11.0, between 11.1–16.6, and greater than or equal to 16.7 mmol/L, respectively [25]. These values may underestimate the severity of hyperglycemia compared with general medical literature, but this would bias our results toward the null hypothesis. Moderate and severe hypoglycemia was defined as a minimum glucose measurement of ≤3.3 and ≤ 2.2 mmol/L, respectively. Glucose measurements below 0.5 mmol/L and above 33.3 mmol/L were considered data entry errors and not included in these calculations.

### Primary outcome

The primary outcome was the occurrence of one or more of the following infectious morbidity events as defined by NSQIP criteria: surgical site infection (SSI), pneumonia, urinary tract infection, or sepsis. Surgical site infections included superficial SSI, deep incisional SSI, organ space SSI, and wound disruption. The standard NSQIP definitions for each of the complications exclude patients with pre-existing infection of that type. (Additional file 2: Appendix 2).

### Statistical analysis

Statistical analysis was performed using SPSS® Version 20 (IBM Inc., Yonkers, New York). Glycemic control was grouped into four categories; normoglycemia, mild hyperglycemia, moderate hyperglycemia, and severe hyperglycemia. Normoglycemia served as the reference group based on the previously stated definitions. In addition, two continuous variables reflecting the glucose standard deviation and GLI were recorded. To adjust for the patient and surgical covariates associated with infectious complications; a multivariable logistic regression model was constructed. Prior to developing the logistic regression model, collinearity diagnostics were performed to ensure that variables were not highly correlated with one another. If the condition index was > 30, then a bivariate Pearson Correlation matrix would be performed to determine which two covariates were highly correlated (> 0.70) [26]. A non-parsimonious logistic regression incorporating covariates and the hyperglycemia groupings, glucose standard deviation and GLI was performed. The covariates used for risk adjustment in this model were: age in years, body mass index (kg/m^2^), male sex, emergency surgery, prolonged operative duration, dyspnea at rest, dyspnea at exertion, new or worsening congestive heart failure, ASA physical status (as a categorical variable), partially dependent functional status, totally dependent functional status, present of ascites, diabetes mellitus requiring oral hypoglycemic therapy, diabetes mellitus requiring insulin therapy, chronic obstructive pulmonary disease, hypertension, angina, history of cardiac surgery, history of percutaneous coronary intervention, cerebrovascular accident, history of transient ischemic attacks, steroid use for a chronic condition, and preoperative serum albumin. All covariates were based upon NSQIP data and dataset definitions (Additional file 1: Appendix 1) and reflect the covariates with the greatest contribution to the NSQIP risk adjustment model [27]. In cases where serum albumin was not available, it was imputed as the median of the patient population. In addition, the underlying risk of each surgical procedure was incorporated using a surgical risk score derived based upon previously described and validated methodology for NSQIP analyses [28]. The derivation dataset for this surgical risk score was the national 2005–2010 NSQIP participant use data file. The surgical risk score is a continuous variable that reflects the underlying risk of the procedure performed, based upon primary common procedural terminology (CPT) codes. The CPT code was developed by the American Medical Association as a way to unify surgical procedures. The codes are broken down into three categories. The highest category is the body region that is being operated on and then can be sub-divided into specific areas within the region or more complex versus less complex surgical techniques. A lower score would indicate the surgical procedure has less risk of post-operative infection and a higher score would indicate the patient is at a greater risk for post-operative infection. Prolonged operative duration is a binary variable based upon the Center for Disease Control’s existing methodology for SSI risk adjustment. It is defined as an operative duration above the 75th percentile for the primary CPT code using national benchmark data. The national benchmark dataset used for this analysis was the 2005–2010 national NSQIP participant use data file. All glucose exposure variables were also included in this model. The goodness of fit of the model was assessed using the Omnibus Tests of Model Coefficients and the Hosmer and Lemeshow Test. All covariates deemed to be significant in the model (*p*-value ≤0.05) were established as independent predictors of an infectious complication. Measures of effect size were reported using the adjusted odds ratio calculated by the logistic regression full model fit [29]. The resulting model’s predictive value was evaluated using the c-statistic for dichotomous outcome [30].

### Sensitivity analysis

A pre-specified sensitivity analysis was performed for all non-diabetic patients using the same non-parsimonious logistic regression techniques and assessment for goodness of fit and the model’s predictive value. Due to the small sample size for diabetic patients, it was not possible to perform a sensitivity analysis due to the model becoming grossly over-fitted.

Two societies have recommended guidelines to target intraoperative glucose under 10 mmol/L for diabetic ambulatory surgical patients and for adult cardiac surgical patients [31, 32]. Therefore, a sensitivity analysis was performed using the same non-parsimonious logistic regression techniques but replacing the previously stated four hyperglycemia groups with a hyperglycemia definition of intraoperative glucose ≥10 mmol/L.

Additionally, intraoperative blood transfusions have been shown to be associated with an increase in complications post-operatively [33]. We performed a sensitivity analysis to incorporate the number of units of red blood cells (RBC) administered intraoperatively to our study cohort to investigate if hyperglycemia and RBC are associated with increased risk of postoperative infectious complications.

### Power analysis

Preliminary data (approximately 14,000 patients) allowed us to ascertain that approximately 30% would include a perioperative glucose measurement and 10% would experience the primary outcome [34]. As a result, we assumed that approximately 300 patients would experience the primary outcome and provide adequate power for a multivariable logistic regression model.

## Results

**Fig. 1:**
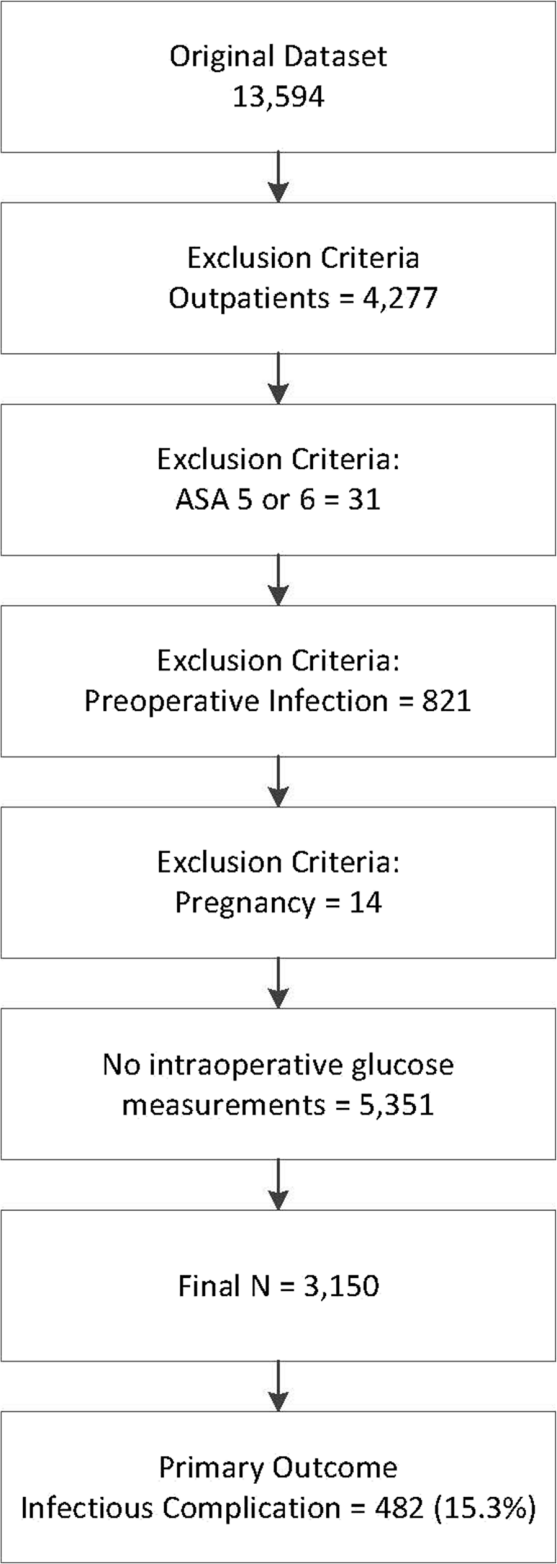
Study selection criteria. *Abbreviations:* ASA = American Society of Anesthesiologists Physical Status classification

13,954 patients with integrated NSQIP and intraoperative EHR were identified. After planned exclusion criteria, 8501 patients undergoing major non-cardiac surgery were identified. Of these, 5351 did not have a perioperative glucose measurement in EHR, resulting in a final analysis dataset of 3150 patients (Fig. 1). A comprehensive listing of procedures included and sample sizes is available in Additional file 3: Appendix 3.

The mean number of measurements per patient was three. 1619 (51%) patients were normoglycemic, 1042 (33%) patients experienced mild hyperglycemia, 442 (14%) experienced moderate hyperglycemia, and 47 (1.5%) experienced severe hyperglycemia. Only 33 patients (1.0%) experienced moderate hypoglycemia while 17 (0.5%) patients experienced severe hypoglycemia (Table 1). Patients experiencing increasing severity of hyperglycemia in general had more comorbidities (Table 2). Overall, 45% of patients demonstrating hyperglycemia did receive intraoperative insulin therapy (32% of mild, 70% of moderate, and 83% of severe hyperglycemia patients). Diabetic patients compared with their non-diabetic counterparts were more likely to demonstrate all levels of hyperglycemia, but not hypoglycemia (Table 1). Overall, 482 (15%) patients experienced a composite postoperative infectious complication (Table 3). The maximum intraoperative glucose distribution for patients with and without the composite postoperative infectious complication as well as the individual infectious complications are shown in Additional file 4: Appendix 4.

**Table 1:** Prevalence of hyperglycemia and hypoglycemia among patients undergoing major non cardiac surgery

	Overall (*N* = 3150) N (%)	Diabetes No (*N* = 2384) N (%)	Diabetes Yes (*N* = 766) N (%)
Normoglycemia (< 8.3 mmol/L)	1619 (51)	1360 (57)	259 (34)
Hyperglycemia			
Mild (8.3–11.0 mmol/L)	1042 (33)	766 (32)	276 (36)
Moderate (11.1–16.6 mmol/L)	442 (14)	244 (10)	198 (26)
Severe (≥16.7 mmol/L)	47 (2.0)	14 (1.0)	33 (4.0)
Hypoglycemia			
Moderate (≤ 3.3 mmol/L)	33 (1.0)	25 (1.0)	8 (1.0)
Severe (≤ 2.2 mmol/L)	17 (0.5)	13 (0.5)	4 (0.5)

**Table 2:** Patient characteristics among studied population

Co-morbidities	Normoglycemia (Glucose < 8.3 mmol/L) (*N* = 1619) N (%)	Mild hyperglycemia (Glucose 8.3–11.0 mmol/L) (*N* = 1042) N (%)	Moderate hyperglycemia (Glucose 11.1–16.6 mmol/L) (*N* = 442) N (%)	Severe hyperglycemia (Glucose ≥16.7 mmol/L) (*N* = 47) N (%)
Age^a^	62 ± 15	63 ± 13	61 ± 14	58 ± 15
Body mass index (kg/m^2^)^a^	28 ± 8	29 ± 8	30 ± 8	31 ± 9
Male sex	935 (58)	588 (56)	229 (52)	23 (49)
Emergency Operation	136 (8.4)	81 (7.8)	43 (9.7)	8 (17)
Dyspnea (any)	174 (11)	115 (11)	58 (13)	7 (15)
Active Congestive Heart Failure	16 (1.0)	9 (0.9)	5 (1.1)	4 (8.5)
ASA^b^ Physical Status 3 or 4	1168 (72)	767 (74)	332 (75)	40 (85)
Functional Status (Partially or Totally Dependent)	53 (3.3)	24 (2.3)	12 (2.7)	2 (4.3)
Ascites	19 (1.2)	7 (0.7)	3 (0.7)	1 (2.1)
Diabetes				
Oral Hypoglycemic Treated	153 (9.5)	177 (17)	112 (25)	12 (26)
Insulin Treated	105 (6.5)	99 (9.5)	86 (20)	21 (45)
COPD^c^	152 (9.4)	89 (8.5)	25 (5.7)	3 (6.4)
Hypertension	956 (59)	613 (59)	278 (63)	31 (66)
Cardiac disease	365 (23)	208 (20)	86 (20)	13 (28)
Cerebrovascular disease	210 (13)	73 (7.0)	47 (11)	3 (6.4)
Renal Failure or Dialysis	53 (3.3)	10 (1.0)	6 (1.4)	2 (4.3)
Operative duration ≥75th percentile for case-specific national norms	566 (35)	470 (45)	222 (50)	27 (59)

^a^Continuous parametric data presented as mean ± standard deviation

^b^ASA = American Society of Anesthesiologists

^c^COPD = Chronic Obstructive Pulmonary Disease

**Table 3:** Primary outcome details

Infection type	Normoglycemia (Glucose < 8.3 mmol/L) (N = 1619) N (%)	Mild hyperglycemia (Glucose 8.3–11.0 mmol/L) (N = 1042) N (%)	Moderate hyperglycemia (Glucose 11.1–16.6 mmol/L) (N = 442) N (%)	Severe hyperglycemia (Glucose ≥16.7 mmol/L) (N = 47) N (%)
Superficial SSI^a^	63 (3.9)	85 (8.2)	29 (6.6)	6 (13)
Deep Incisional SSI^a^	14 (0.9)	15 (1.4)	3 (0.7)	0 (0)
Organ space SSI^a^	30 (1.9)	32 (3.1)	20 (4.5)	0 (0)
Wound disruption	10 (0.6)	9 (0.9)	6 (1.4)	2 (4.3)
Pneumonia	36 (2.2)	29 (2.8)	17 (3.8)	0 (0)
Urinary Tract Infection	69 (4.3)	48 (4.6)	23 (5.2)	3 (6.4)
Sepsis	45 (2.8)	45 (4.3)	21 (4.8)	0 (0)

^a^SSI = Surgical Site Infection

Note: Some patients experienced more than one infection

**Fig. 2:**
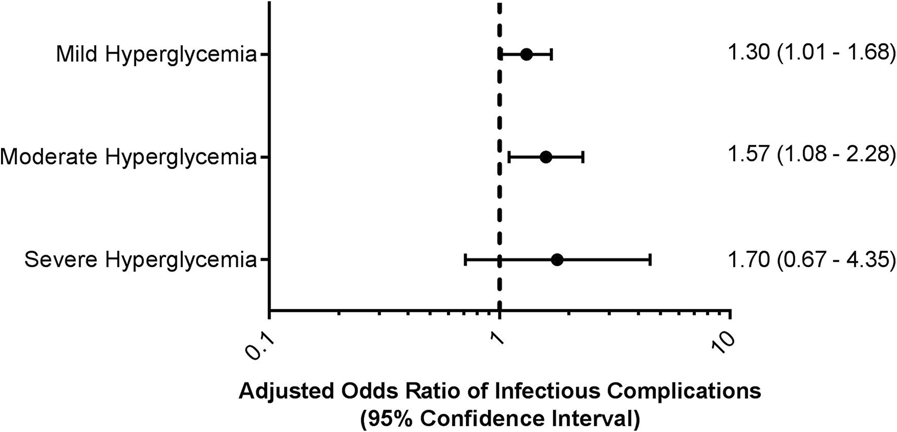
Risk-adjusted increase in infectious complications for hyperglycemia patients

Covariate adjusted logistic regression analysis included 3036 patients (96%) with complete data. Compared with normoglycemic controls, patients with mild hyperglycemia had a statistically significant risk-adjusted increase in infectious complications (adjusted odds ratio (AOR)1.30 [95% confidence interval (CI) 1.01 to 1.68], *p*-value = 0.04). Compared with normoglycemic controls, patients with moderate hyperglycemia also had a statistically significant risk-adjusted increase in infections complications (AOR 1.57 [95% CI 1.09–2.28], *p*-value = 0.02) (Fig. 2). Severe hyperglycemia, glucose standard deviation, and GLI did not demonstrate a statistically significant independent relationship. Additional covariates with an independent association to the primary infectious outcome were: male sex (protective), dyspnea at moderate exertion, functional status (both partially and totally dependent), preoperative serum albumin level, chronic obstructive pulmonary disease, prolonged operative duration, and surgical risk score. The model’s c-statistic was 0.72 [95% CI 0.69–0.74] and the omnibus test of model coefficients resulting in a chi-square of 242.0, 29 degrees of freedom, and *p*-value < 0.001. The Hosmer and Lemeshow Test resulted in a chi-square of 3.3, 8 degrees of freedom, and a p-value of 0.92.

When studying the subset of patients without diabetes, mild hyperglycemia, moderate hyperglycemia, severe hyperglycemia, glucose standard deviation, and GLI did not demonstrate a significant independent relationship. The model’s c-statistic was 0.71 [95% CI 0.69–0.74] and the omnibus test of model coefficients resulted in a chi-square of 195.9, 27 degrees of freedom, and a *p*-value< 0.001. The Hosmer and Lemeshow Test resulted in a chi-square of 3.0, 8 degrees of freedom, and a p-value of 0.93.

Classification of hyperglycemia defined as a glucose value ≥10 mmol/L did not demonstrate a statistically significant risk adjusted increase in postoperative infectious complications (AOR 1.06 [95% CI 0.80–1.40]). Our sensitivity analysis on the use of RBC intraoperatively demonstrated similar measures of effect sizes for the hyperglycemia groupings (data not shown) and in addition a statistically significant measure of effect size for the number of RBC units administered [[AOR 1.04 (95% CI 1.00–1.07)].

## Discussion

Our data demonstrate that among a broad range of patients undergoing major non-cardiac surgery, intraoperative glucose levels above 8.3 mmol/L are associated with a significantly increased risk of postoperative infections. This effect is independent of underlying patient comorbidities, surgical procedure complexity, and surgical duration. A dose-response curve was observed, with mild hyperglycemia resulting in an AOR of 1.30 [95% CI of 1.01–1.68] and moderate hyperglycemia resulting in an AOR of 1.57 [95% CI of 1.08–2.28]. Although the data were underpowered to detect a statistically significant increase among patients with severe hyperglycemia, an AOR of 1.70 [95% CI of 0.67–4.35] was observed. This analysis of 3150 patients is the largest published dataset of intraoperative glucose management and serves to inform future prospective interventional studies.

There are very limited prospective data evaluating the impact of intraoperative hyperglycemia and postoperative outcomes in the non-cardiac surgery population [1]. A recent randomized controlled trial limited to 236 patients undergoing major vascular surgery did find a decreased risk of cardiac adverse events in patients targeted to a glucose level below 8.3 mmol/L, but the patient population and outcome of interest were limited [16]. The primary outcome was a composite outcome of all-cause mortality and two specific cardiac events; myocardial infarction and acute congestive heart failure. Infectious complications were not evaluated. Next, secondary interim analyses of 381 patients enrolled in the Dexamethasone, Light Anesthesia and Tight Glucose Control (DeLiT) trial did not demonstrate a relationship between intensive insulin therapy and a composite morbidity and mortality endpoint [17].

Even observational trials struggle to offer insight into associations between intraoperative glucose values and postoperative outcomes. Of the many observational studies evaluating glucose control and postoperative outcomes, few have been in the non-cardiac literature, [10, 12–15, 30] and only two have incorporated intraoperative glucose measurements into the analysis [11, 35]. Eshuis et al. reviewed the glycemic control of 330 patients undergoing elective pancreatoduodenectomy [11]. They did not detect any relationship between intraoperative hyperglycemia and a composite morbidity outcome, but were likely underpowered to detect a difference with only 330 patients. Yoo et al. retrospectively reviewed 304 patients undergoing liver transplantation and demonstrated that glucose variability increased a patient’s risk for postoperative acute kidney injury but not hyperglycemia alone. Our study did include glucose variability in the multivariate modeling but that was not determined to be an independent predictor of postoperative infectious complications [35].

Our data demonstrate that the risk-adjusted association between hyperglycemia and postoperative infections is present with increasing severity of hyperglycemia resulting in an increased risk of infectious complications after adjusting for surgical complexity and patient pre-existing comorbidities. From a pathophysiologic viewpoint, hyperglycemia is associated with increased inflammation, impaired chemotaxis and phagocytosis, and vulnerability to infections [1, 6]. The cardiac surgery literature has extensive observational and some prospective randomized trial data to support the relationship between improved glycemic control and decreased surgical site infections [1, 6]. Though our data demonstrates an association between intraoperative hyperglycemia and postoperative infections in patients undergoing non-cardiac surgeries, the causal relationship still remains unclear.

There is a lack of published guidelines regarding intraoperative glucose management in the diabetic or non-diabetic patient undergoing non-cardiac surgeries. The results of this study highlights the significance of intraoperative glucose levels and in addition questions the practice pattern of casually dismissing an intraoperative glucose measurement of 10 mmol/L as some medical societies have recommended [31, 32]. We have demonstrated that when a binary threshold for hyperglycemia defined as a glucose ≥10 mmol/L is used, there is no significant effect size measures associated with postoperative infections. However, in contrast, when three separate ranges of intraoperative hyperglycemia levels are used, a dose-response curve is seen. These data may suggest that more patients undergoing major non-cardiac surgery warrant intraoperative glucose measurement. At the very least, intraoperative hyperglycemia warrants a discussion with the surgical team to inform them of the patient’s elevated risk of postoperative infections. In this dataset, more than half of patients undergoing major non-cardiac surgery did not have an intraoperative glucose measurement (Fig. 1). In general, these patients were healthier and less likely to have a preoperative diagnosis of diabetes (data not shown). It should be noted that the observed relationship between hyperglycemia and increased infectious complications was observed in the primary analysis and our sensitivity analysis incorporating the number of RBC units administered intraoperatively. Glucose measurement and management must broaden its scope from the diabetic patient undergoing non-cardiac surgery to all patients including non-DM patients undergoing major surgery.

There are several limitations to this study. As an observational study, an association between intraoperative hyperglycemia and postoperative infection was demonstrated, but causal relationship could not be established. Despite adjusting for a variety of comorbidities and surgical factors, residual confounding may be present. Several factors that impact infectious complications, such as antibiotic choice, normothermia, and pre-existing immunity deficient states (HIV/AIDS and disseminated cancer) were not incorporated into the analysis. In our database, only 4 patients were diagnosed with HIV/AIDS. This sample size is very small and will not affect the measures of effect sizes on mild and moderate hyperglycemia demonstrated in our primary analysis. We also have 155 patients (4.9%) with a preoperative diagnosis of disseminated cancer. Hyperglycemia, like a suppressed immune system, are only modifiers of susceptibility to infections. Therefore, we did not exclude those patients from the database. Next, the analysis does not address the effect of hyperglycemia treatment. Although many patients did receive intraoperative insulin treatment and these data are reported, the logistic regression did not include insulin treatment as a covariate because insulin treatment is a marker of hyperglycemia itself. Larger observational studies are needed to compare patients with similar hyperglycemia profiles with and without insulin treatment to ascertain an insulin treatment effect. Until then the real risks of hyperglycemia must be weighed against the independent relationship that we observed between intraoperative hyperglycemia and postoperative infection. The point estimates for hyperglycemia may demonstrate a dose response curve, however the wide confidence interval for severe hyperglycemia may indicate it’s not. In addition, the NSQIP definitions of SIRS, sepsis, and septic shock have been enhanced recently. Unfortunately, we do not have the specific data elements available (positive blood culture, clinical documentation or purulence, or suspected pre-operative clinical condition or infection or bowel infarction) that are included in the new definition to adequately perform a sensitivity analysis. At our institution, there are no differences in rates of prophylactic antibiotic administration. These data were collected using internal quality assurance metrics and are unable to be linked to this database to allow a propensity score matched analysis to address any potential confounding between prophylactic antibiotic administration and infectious outcomes. It is feasible, that our sample size in the study cohort would be reduced and the dose response curve in intraoperative hyperglycemia and postoperative infectious complications may differ. Although these data add to the non-cardiac literature, the patient population is limited to major general, vascular, and urologic surgery; other common procedures warrant further investigation. The single-center nature of the dataset also limits the generalizability of the conclusions. The data, due to low sample size, were also underpowered to detect a difference among patients experiencing severe hyperglycemia and a sensitivity analysis focused on diabetic patients only was not feasible. Therefore, future clinical trials are needed to focus on diabetic patients. We also acknowledge that the overall composite infection rate is high (15%) in relation to previously published research. However, the individual infection outcomes (Table 3) have an overall incidence ranging from 0.9–5.8%. NSQIP methodology ensures high quality surgical clinical reviewer extraction for all data elements with less than 2% disagreement in inter-rater reliability audit checks.

## Conclusions

Despite these limitations, these data are impactful and novel. More than 3000 patients undergoing a variety of major non-cardiac surgeries were studied, representing the largest observational dataset regarding intraoperative hyperglycemia in non-cardiac surgery to date. The data clearly demonstrate a dose-dependent relationship between intraoperative hyperglycemia and postoperative infectious complications after risk adjustment for major comorbidities. Intraoperative hyperglycemia is an independent predictor of infectious complications among both diabetic and non-diabetic patients and must become a focus among practicing anesthesiologists. These data demand further exploration beyond infectious complication via larger, multicenter observational datasets and prospective, randomized control trials to establish whether treatment of hyperglycemia may alter outcomes. Non diabetic patients and impact of glucose variability is worth exploring including the value of commercially available continuous glucose monitoring.

## Additional files

Additional file 1: Appendix 1. American College of Surgeons - National Surgery Quality Improvement Program Data Element Definitions. (DOC 44 kb)

Additional file 2: Appendix 2. American College of Surgeons – National Surgery Quality Improvement Program Data Outcome Definitions. (DOC 58 kb)

Additional file 3: Appendix 3. Frequency and Infectious Complication Data by Procedure Description. (DOCX 18 kb)

Additional file 4: Appendix 4. Intraoperative Maximum Glucose Distribution for Patients With and Without Postoperative Infectious Complications. Box and whisker plots for maximum intraoperative blood glucose value (mmol/L) by overall complication infectious complication as well as individual infectious complications. *Abbreviations*: SSI = Surgical Site Infection, UTI = Urinary Tract Infection. (TIF 381 kb)

## Abbreviations

AOR: Adjusted Odds Ratio
ASA: American Society of Anesthesiologists
CI: Confidence Interval
COPD: Chronic Obstructive Pulmonary Disease
CPT: Common Procedural Terminology
EHR: Electronic Health Record
GLI: Glycemic Lability Index
IIT: Intensive Insulin Therapy
NSQIP: National Surgical Quality Improvement Program
SSI: Surgical Site Infection
