# Supplementary material for: Intraoperative hyperglycemia is independently associated with infectious complications after non-cardiac surgery
Source: BMC Anesthesiol. 2018 Jul 19;18:90. doi: 10.1186/s12871-018-0546-0 (PMC6053803; doi:10.1186/s12871-018-0546-0)
Supplement: Supplementary file 1 — American College of Surgeons - National Surgery Quality Improvement Program Data Element Definitions. (DOC 44 kb) [file 12871_2018_546_MOESM1_ESM.doc]

Appendix 1: American College of Surgeons – National Surgery Quality Improvement Program Data element definitions

| Data element | Definition |
| --- | --- |
| Emergency surgery | An emergency case is usually performed as soon as possible and no later than 12 hours after the patient has been admitted to the hospital or after the onset of related preoperative symptomatology. Answer ‘yes’ if the surgeon and anesthesiologist report the  case as emergent |
| Dyspnea with moderate exertion | Patient is unable to climb one flight of stairs without shortness of breath. |
| Dyspnea at rest | The patient cannot complete a sentence without needing to take a breath. |
| Active congestive heart failure | Congestive heart failure is the inability of the heart to pump a sufficient quantity of blood to meet the metabolic needs of the body or can do so only at increased ventricular filling pressure. Only newly diagnosed CHF within the previous 30 days or a diagnosis of chronic CHF with new signs or symptoms in the 30 days prior to surgery fulfills this definition. Common manifestations are:  - Abnormal limitation in exercise tolerance due to dyspnea or fatigue  - Orthopnea (dyspnea on lying supine)  - Paroxysmal nocturnal dyspnea (awakening from sleep with dyspnea)  - Increased jugular venous pressure  - Pulmonary rales on physical examination  - Cardiomegaly  - Pulmonary vascular engorgement  Should be noted in the medical record as CHF, congestive heart failure, or pulmonary edema |
| Functional status independent | The patient does not require assistance from another person for any activities of daily living. This includes a person who is able to function independently with prosthetics, equipment, or devices. |
| Functional status partially dependent | The patient requires some assistance from another person for activities of daily living. This includes a person who utilizes prosthetics, equipment, or devices but still requires some assistance from another person for activities of daily living. |
| Functional status totally dependent | The patient requires total assistance for all activities of daily living. |
| Ascites | The presence of fluid accumulation in the  peritoneal cavity noted on physical examination, abdominal ultrasound, or abdominal  CT/MRI within 30 days prior to the operation. |
| Orally controlled diabetes mellitus | Report the treatment regimen of the patient’s chronic, long-term management. Do not include a patient if diabetes is controlled by diet alone. A diagnosis of diabetes requiring therapy with an oral hypoglycemic agent  Insulin: a diagnosis of diabetes requiring daily insulin therapy |
| Insulin controlled diabetes mellitus | Report the treatment regimen of the patient’s chronic, long-term management. Do not include a patient if diabetes is controlled by diet alone. A diagnosis of diabetes requiring daily insulin therapy |
| History of chronic obstructive pulmonary disease (COPD) | Chronic obstructive pulmonary disease (such as emphysema and/or chronic bronchitis) resulting in any one or more of the following:  - Functional disability from COPD (e.g., dyspnea, inability to perform activities of daily living)  - Hospitalization in the past for treatment of COPD  - Requires chronic bronchodilator therapy with oral or inhaled agents  - An Forced Expiratory Volume of <75% of predicted on pulmonary function testing  Do not include patients whose only pulmonary disease is asthma, an acute and chronic  inflammatory disease of the airways resulting in bronchospasm. Do not include patients with  diffuse interstitial fibrosis or sarcoidosis. |
| Hypertension | The patient has a persistent elevation of systolic  blood pressure > 140 mmHg **or** a diastolic blood pressure > 90 mmHg **or** requires an antihypertensive treatment (e.g., diuretics, beta blockers, ACE inhibitors, calcium channel  blockers) at the time the patient is being considered as a candidate for surgery (which should be no longer than 30 days prior to surgery). Hypertension must be documented in the patient’s chart. |
| Cardiac disease | **Percutaneous coronary intervention**  The patient has undergone percutaneous coronary intervention at any time (including any attempted intervention). This includes either balloon dilatation or stent placement. This does not include valvuloplasty procedures  OR  **Previous cardiac surgery**  Any major cardiac surgical procedure (performed either as an ‘off-pump’ repair or utilizing cardiopulmonary bypass). This includes coronary artery bypass graft surgery, valve replacement or repair, repair of atrial or ventricular septal defects, great thoracic vessel repair, cardiac transplant, left ventricular aneurysmectomy, insertion of left ventricular assist devices, etc. Do not include pacemaker insertions or automatic implantable cardioverter defibrillator insertions.  OR  **Angina within 1 month of surgery**  Pain or discomfort between the  diaphragm and the mandible resulting from myocardial ischemia. Typically angina is a dull,  diffuse (fist-sized or larger) substernal chest discomfort precipitated by exertion or emotion  and relieved by rest or nitroglycerine. Radiation to the arms and shoulders often occurs, and  occasionally to the neck, jaw (mandible, not maxilla), or interscapular region. Documentation in the chart by the physician should state ‘angina’ or ‘anginal equivalent’. For patients on antianginal medications, enter ‘yes’ *only* if the patient has had angina at any time within one  month prior to surgery.  OR  **Myocardial infarction within six months**  The history of a non-Q wave or a Q wave infarct in the six months prior to surgery as diagnosed in the patient’s medical record |
| Cerebrovascular disease | **History of transient ischemic attacks:** Transient ischemic attacks are focal neurologic deficits (e.g. numbness of an arm or amaurosis fugax) of sudden onset and brief duration (usually <30 minutes) that usually reflects dysfunction in a cerebral vascular distribution. These attacks may be recurrent and, at times, may precede a stroke.  OR  **CVA/Stroke with or without neurological deficit:** History of a cerebrovascular accident (embolic, thrombotic, or hemorrhagic) lasting at least 30 minutes with or without persistent residual motor, sensory, or cognitive dysfunction. |
| Acute renal failure | The clinical condition associated with rapid, steadily increasing azotemia (increase in BUN) and a rising creatinine of above 3 mg/dl. Acute renal failure should be noted within 24 hours prior to surgery |
| Preoperative dialysis dependence | Acute or chronic renal failure requiring treatment with peritoneal dialysis, hemodialysis, hemofiltration, hemodiafiltration, or ultrafiltration within 2 weeks prior to surgery. |
| Steroid use for chronic condition | Patients who required regular administration of oral or parenteral corticosteroid medication in the 30 days prior to surgery for a chronic medical condition. Patients on a short course (<10 days) are not included. |
| Cancer | Disseminated cancer |
