# Supplementary material for: Intraoperative hyperglycemia is independently associated with infectious complications after non-cardiac surgery
Source: BMC Anesthesiol. 2018 Jul 19;18:90. doi: 10.1186/s12871-018-0546-0 (PMC6053803; doi:10.1186/s12871-018-0546-0)
Supplement: Supplementary file 2 — American College of Surgeons – National Surgery Quality Improvement Program Data Outcome Definitions. (DOC 58 kb) [file 12871_2018_546_MOESM2_ESM.doc]

Appendix 2: American College of Surgeons – National Surgery Quality Improvement Program Data outcome definitions

| Data element | Definition |
| --- | --- |
| Superficial Incisional Surgical Site Infection (SSI) | Superficial incisional SSI is an infection that occurs within 30 days after the operation *and* infection involves only skin or subcutaneous issue of the incision *and* at least *one* of the following:   - Purulent drainage, with or without laboratory confirmation, from the superficial incision. - Organisms isolated from an aseptically obtained culture of fluid or issue from the superficial incision. - At least one of the following signs or symptoms of infection: pain or tenderness, localized swelling, redness, or heat AND superficial incision is deliberately opened by the surgeon, unless incision is culture-negative. - Diagnosis of superficial incisional SSI by the surgeon or attending physician.   *Do not report the following conditions of SSI:*   - Stitch abscess (minimal inflammation and discharge confined to the points of suture penetration). - Infected burn wound. - Incisional SSI that extends into the fascial and muscle layers (see deep incisional SSI). |
| Deep Incisional SSI | Deep Incision SSI is an infection that occurs within 30 days after the  operation and the infection appears to be related to the operation *and* infection involved deep soft tissues (e.g., fascial and muscle layers) of the incision *and* at least *one* of the following:   - Purulent drainage from the deep incision but not from the organ/space component of the surgical site. - A deep incision spontaneously dehisces or is deliberately opened by a surgeon when the patient has at least one of the following signs or symptoms: fever (> 38 C), localized pain, or tenderness, unless site is culture-negative. - An abscess or other evidence of infection involving the deep incision is found on direct examination, during reoperation, or by histopathologic or radiologic examination. - Diagnosis of a deep incision SSI by a surgeon or attending physician.   *Note:*   - Report infection that involves both superficial and deep incision sites as deep incisional SSI. - Report an organ/space SSI that drains through the incision as a deep incisional SSI. |
| Organ Space SSI | Organ/Space SSI is an infection that occurs within 30 days after the operation and the infection appears to be related to the operation *and* the infection involves any part of the anatomy (e.g., organs or spaces), other than the incision, which was opened or manipulated during an operation *and* at least *one* of the following:   - Purulent drainage from a drain that is placed through a stab wound into the organ/space. - Organisms isolated from an aseptically obtained culture of fluid or tissue in the organ/space. - An abscess or other evidence of infection involving the organ/space that is found on direct examination, during reoperation, or by histopathologic or radiologic examination. - Diagnosis of an organ/space SSI by a surgeon or attending physician. |
| Wound disruption | Separation of the layers of a surgical wound, which may be partial or complete, with disruption of the fascia. |
| Pneumonia | Inflammation of the lungs caused primarily by bacteria, viruses, and/or chemical irritants, usually manifested by chills, fever, pain in the chest, cough, purulent, bloody sputum. Enter "YES" if the patient has pneumonia meeting the definition of pneumonia below **AND** pneumonia not present preoperatively.  Pneumonia must meet one of the following TWO criteria:  ***Criterion 1*:** Rales or dullness to percussion on physical examination of chest AND any of the following:   1. New onset of purulent sputum or change in character of sputum 2. Organism isolated from blood culture 3. Isolation of pathogen from specimen obtained by transtracheal aspirate, 4. Bronchial brushing, or biopsy   **OR**  ***Criterion 2:*** Chest radiographic examination shows new or progressive infiltrate, consolidation, cavitation, or pleural effusion AND any of the following:   1. New onset of purulent sputum or change in character of sputum 2. Organism isolated from blood culture 3. Isolation of pathogen from specimen obtained by transtracheal aspirate, bronchial brushing, or biopsy 4. Isolation of virus or detection of viral antigen in respiratory secretions 5. Diagnostic single antibody titer (IgM) or fourfold increase in paired serum samples (IgG) for pathogen 6. Histopathologic evidence of pneumonia |
| Urinary Tract Infection | Postoperative symptomatic urinary tract infection must meet one  of the following TWO criteria:   1. *One* of the following:  - fever (>38 degrees C) - urgency - frequency - dysuria - suprapubic tenderness   *AND* a urine culture of > 10^5^ colonies/ml urine with no more than two species of organisms  **OR**   1. *Two* of the following:  - fever (>38 degrees C) - urgency - frequency - dysuria - suprapubic tenderness   *AND* any of the following:   - Dipstick test positive for leukocyte esterase and/or nitrate - Pyuria (>10 WBCs/cc or > 3 WBC/hpf of unspun urine) - Organisms seen on Gram stain of unspun urine - Two urine cultures with repeated isolation of the same uropathogen with >10^2^ colonies/ml urine in non-voided specimen - Urine culture with < 10^5^ colonies/ml urine of single uropathogen in patient being treated with appropriate antimicrobial therapy - Physician's diagnosis - Physician institutes appropriate antimicrobial therapy |
| Sepsis | Sepsis is the systemic response to infection. Report this variable if the  patient has two of the following clinical signs and symptoms of SIRS:   - Temp >38 degrees C or <36 degrees C - Heart rate >90 beats per minute - Respiratory Rate >20 breaths/min or PaCO2 <32 mmHg(<4.3 kPa) - WBC >12,000 cell/mm3, <4000 cells/mm3, or >10% immature (band) forms - Anion gap acidosis: this is defined by either: - [Na + K] – [Cl + HCO3 (or serum CO2)]. If this number is greater than 16, then an anion gap acidosis is present. - Na – [Cl + HCO3 (or serum CO2)]. If this number is greater than 12, then an anion gap acidosis is present.   **and** one of the following:   - positive blood culture - clinical documentation of purulence or positive culture from any site thought to be causative |
