# Supplementary material for: Intraoperative hyperglycemia is independently associated with infectious complications after non-cardiac surgery
Source: BMC Anesthesiol. 2018 Jul 19;18:90. doi: 10.1186/s12871-018-0546-0 (PMC6053803; doi:10.1186/s12871-018-0546-0)
Supplement: Supplementary file 3 — Frequency and Infectious Complication Data by Procedure Description. (DOCX 18 kb) [file 12871_2018_546_MOESM3_ESM.docx]

**Appendix 3**

| Procedure Description | Common Procedural Terminology Codes Included | Frequency (n) | Infectious Complication  n (%) |
| --- | --- | --- | --- |
| Vascular Surgery involving the carotid/vertebral or any artery | 35001, 35201, 35301, 35506, 35510, 35601, 35606, 35626, 35691, 35694, 35800, 60600 | 308 | 7 (2.3) |
| Hepatobiliary – Pancreas debridement or resection | 48005, 48120, 48140, 48145, 48150, 48152, 48153, 48154, 48155, 48180, 48999 | 281 | 67 (24) |
| Colorectal – Open Colectomy,Colostomy, or Stoma | 44140, 44141, 44143, 44144, 44145, 44146, 44150, 44152, 44153, 44155, 44158, 44160, 44320, 44340, 44604, 44620, 44625, 44626 | 227 | 59 (26) |
| Vascular Surgery – Open abdominal aorta aneurysm (AAA) repair and Iliac | 34830, 34831, 35081, 35082, 35091, 35092, 35102, 35103, 35131, 35351, 35361, 35531, 35631, 35531, 35637, 35638, 35641, 35646, 35647, 35840 | 220 | 20 (9.1) |
| Hepatobiliary Liver Resection | 47120, 47122, 47125, 47130 | 147 | 25 (17) |
| Vascular Surgery – Endovascular AAA | 34800, 34802, 34803, 34804, 34805, 34825 | 137 | 7 (5.1) |
| Vascular Surgery – Chest/Extremity – Lower extremity Artery Thrombectomy/Repair | 34151, 34201, 34203, 35141, 35142, 35151, 35226, 35256, 35286, 35302, 35371, 35372, 35761, 35879, 35881, 35883 | 118 | 9 (7.6) |
| Vascular Surgery – Chest/Extremity – Lower extremity Artery Bypass | 34900, 35556, 35558, 35566, 35571, 35583, 35585, 35656, 35661, 35665, 35666, 35671 | 118 | 9 (7.6) |
| Small Intestine – Open Resection/Ostomy | 44120, 44125, 44130, 44310, 44314 | 89 | 31 (35) |
| Genitourinary – Bladder Procedure | 51590, 51595, 51596, 51865 | 85 | 19 (22) |
| Genitourinary – open or partial nephrectomy | 50220, 50225, 50230, 50234, 50240 | 66 | 8 (12) |
| Musculoskeletal – Lower extremity amputation | 27295, 27590, 27592, 27596, 27880, 27882, 27886, 28800, 28805 | 59 | 12 (20) |
| Esophago-gastric – Major open gastric resection | 43621, 43622, 43631, 43632, 43633, 43820, 43860 | 57 | 12 (21) |
| Abdominal – Open exploration | 49000, 49002, 49900, 49999 | 56 | 15 (27) |
| Genitourinary – laparoscopic (including partial) nephrectomy | 50543, 50545, 50546, 50548 | 55 | 3 (5.5) |
| Abdominal – Intra-abdominal/retroperitoneal tumor resection | `49010, 49200, 49201, 49203, 49204, 49205, 49215 | 52 | 9 (17) |
| Vascular – Abdominal vascular (non Aorta), Arterial | 35111, 35121, 35221, 35251, 35281, 35560, 37617 | 52 | 8 (15) |
| Head and Neck – Thyroid tumor | 60210, 60220, 60240, 60252, 60260, 60270, 60271 | 49 | 2 (4.1) |
| Hernia – Open ventral, umbilical, incisional or other | 49560, 49561, 49565, 49566, 49568, 49585, 49587 | 46 | 7 (15) |
| Hepatobiliary – Biliary Tree, open cholecystectomy | 47600, 47605, 47610, 47612 | 45 | 7 (16) |
| Genitourinary – Open Prostrate/Urethra | 55810, 55840, 55845 | 45 | 4 (8.9) |
| Colorectal – Open abdominal-perineal resection | 45110, 45111, 45113, 45119, 45136 | 42 | 9 (21) |
| Small Intestine – Open, no resection/Lysis of adhesions /Incision | 44005, 44010, 44025, 44050, 44110, 44111, 44312, 44602, 44603 | 41 | 8 (20) |
| Hepatobiliary – Liver, no resection | 47300, 47379, 47380 | 41 | 4 (9.8) |
| Hepatobiliary – Biliary Tree, Open-complex reconstruction | 47420, 47711, 47760, 47765, 47780, 47785, 47800, 47900 | 38 | 12 (32) |
| Hepatobiliary – Biliary Tree, laparoscopic cholecystectomy | 47562, 47563 | 37 | 5 (14) |
| Adrenal – Laparoscopic Adrenalectomy | 60650 | 36 | 1 (2.8) |
| Skin and Soft Tissue – Lymph Biopsy,Dissection, or removal | 38308, 38542, 38562, 38724, 38745, 38760, 38770, 38780 | 30 | 3 (10) |
| Head and Neck – Parayhyroid tumor | 60500, 60502 | 30 | 1 (3.3) |
| Vascular – Chest/Extremity, Upper extremity chest artery thrombectomy or repair | 34101, 35011, 35021, 35045, 35190, 35206, 35236, 35860, 35875, 35876, 37618 | 29 | 4 (14) |
| Colorectal – Any bowel fistula management | 44640, 44660, 44661, 45800, 45820, 57288 | 23 | 7 (30) |
| Spleen – Open splenectomy or splenorrhaphy | 38100, 38102 | 23 | 2 (8.7) |
| Vascular – Thoracic or ascending Aorta or Iliac | 33863, 33875, 33877 | 23 | 8 (35) |
| Breast – Excisional mastectomy, including partial | 19160, 19180, 19240, 19303, 19307 | 19 | 1 (5.3) |
| Vascular – Endovascular thoracoabdominal repair of Aorta | 33880, 33881 | 17 | 1 (5.9) |
| Reconstructive Surgery – Any flap | 14300, 15734, 15738 | 15 | 7 (47) |
| Genitourinary – Laparoscopic prostate or urethra | 55866 | 15 | 0 (0) |
| Colorectal – Laparoscopic colectomy, colostomy, or stoma | 44188, 44204, 44205, 44227 | 14 | 4 (29) |
| Genitourinary - Ureter | 50780, 50800, 50820, 50825, 50830 | 11 | 3 (27) |
| Other | 11043, 13102, 15100, 21620, 27680, 33120, 33335, 34808, 35002, 35188, 35231, 35261, 35276, 35508, 355515, 35616, 35632, 35633, 35636, 35645, 35695, 37160, 37187, 37207, 39501, 43246, 49320, 49904, 50010, 50727, 50783, 50785, 50949, 51050, 51575, 51597, 51702, 51960, 52000, 52001, 52005, 52281, 52282, 52310, 52330, 52332, 54125, 55500, 58559, 64577, 97606 | 119 | 38 (32) |
