# Supplementary figures and images for: Intraoperative hyperglycemia is independently associated with infectious complications after non-cardiac surgery
Source: BMC Anesthesiol. 2018 Jul 19;18:90. doi: 10.1186/s12871-018-0546-0 (PMC6053803; doi:10.1186/s12871-018-0546-0)

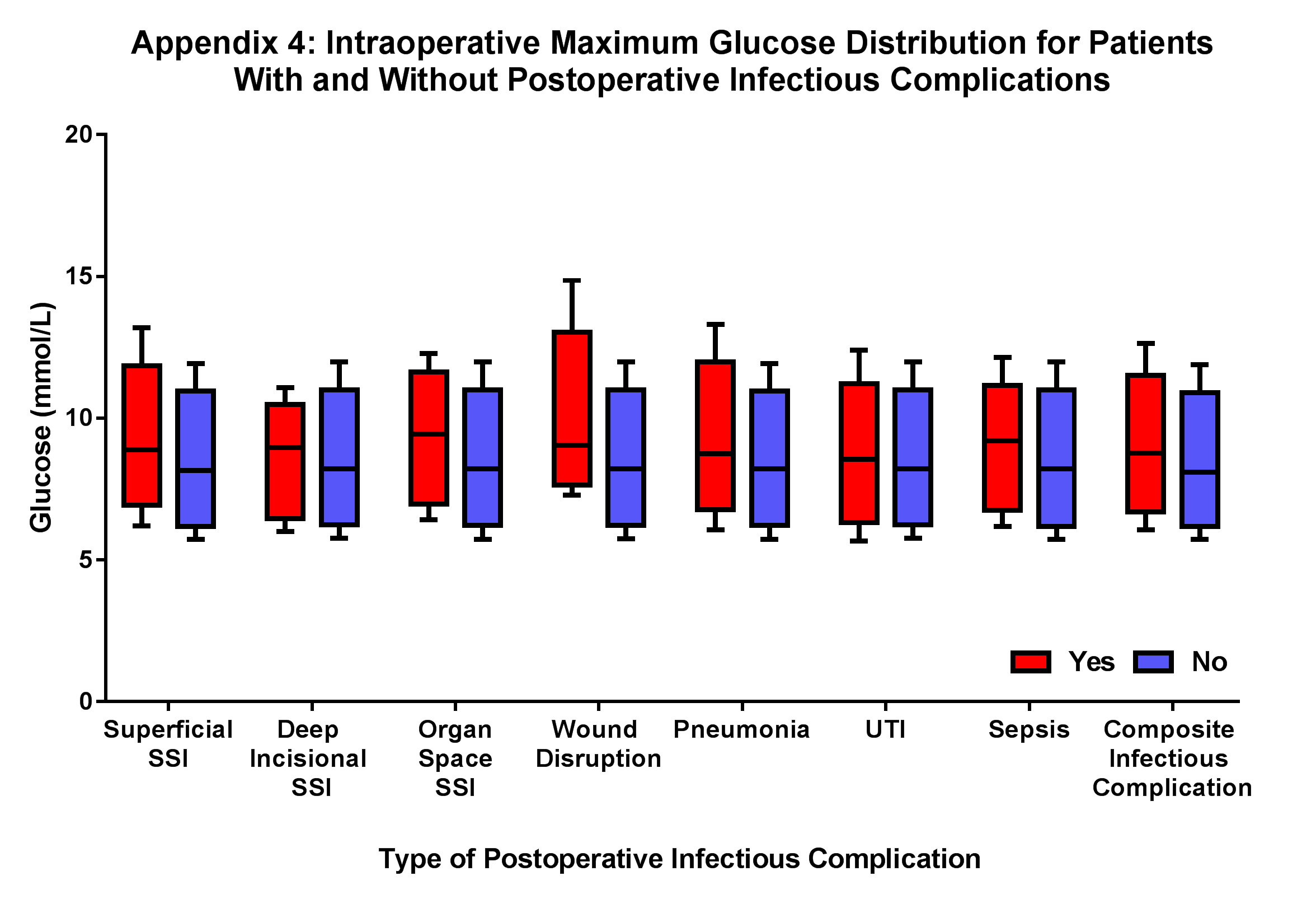

Supplement: Supplementary file 4 — Intraoperative Maximum Glucose Distribution for Patients With and Without Postoperative Infectious Complications. Box and whisker plots for maximum intraoperative blood glucose value (mmol/L) by overall complication infectious complication as well as individual infectious complications. Abbreviations: SSI = Surgical Site Infection, UTI = Urinary Tract Infection. (TIF 381 kb) [file 12871_2018_546_MOESM4_ESM.tif]
